# Not so free range? Oviposition microhabitat and egg clustering affects *Eretmoptera murphyi* (Diptera: Chironomidae) reproductive success

**DOI:** 10.1007/s00300-018-2420-4

**Published:** 2018-10-16

**Authors:** Jesamine Bartlett, Pete Convey, Scott A. L. Hayward

**Affiliations:** 1 University of Birmingham, Edgbaston, Birmingham, B15 2TT UK; 2 British Antarctic Survey, NERC, High Cross, Madingley Rd, Cambridge, CB3 0ET UK

**Keywords:** Desiccation, Heat tolerance, Parthenogenesis, Life history, Antarctica, Climate change, Invasive species

## Abstract

Understanding the physiology of non-native species in Antarctica is key to elucidating their ability to colonise an area, and how they may respond to changes in climate. *Eretmoptera murphyi* is a chironomid midge introduced to Signy Island (Maritime Antarctic) from South Georgia (Sub-Antarctic) where it is endemic. Here, we explore the tolerance of this species’ egg masses to heat and desiccation stress encountered within two different oviposition microhabitats (ground surface vegetation and underlying soil layer). Our data show that, whilst oviposition takes place in both substrates, egg sacs laid individually in soil are at the greatest risk of failing to hatch, whilst those aggregated in the surface vegetation have the lowest risk. The two microhabitats are characterised by significantly different environmental conditions, with greater temperature fluctuations in the surface vegetation, but lower humidity (%RH) and available water content in the soil. Egg sacs were not desiccation resistant and lost water rapidly, with prolonged exposure to 75% RH affecting survival for eggs in singly oviposited egg sacs. In contrast, aggregated egg sacs (*n *= 10) experienced much lower desiccation rates and survival of eggs remained above 50% in all treatments. Eggs had high heat tolerance in the context of the current microhabitat conditions on Signy. We suggest that the atypical (for this family) use of egg sac aggregation in *E. murphyi* has developed as a response to environmental stress. Current temperature patterns and extremes on Signy Island are unlikely to affect egg survival, but changes in the frequency and duration of extreme events could be a greater challenge.

**Electronic supplementary material:**

The online version of this article (10.1007/s00300-018-2420-4) contains supplementary material, which is available to authorized users.

## Introduction

*Eretmoptera murphyi* (Schaeffer 1914) (Chironomidae, Orthocladiinae) is a flightless midge endemic to the sub-Antarctic island of South Georgia (54°S, 36°W), from where it was introduced to Signy Island (South Orkney Islands, maritime Antarctic 60°43′0″S,45°36′0″W) in the 1960s (Block et al. 1984; Convey and Block 1996). It is one of Antarctica’s few holometabolous insects, and the only macro terrestrial invertebrate and true insect found on Signy Island (Convey and Block 1996; Chown and Convey 2016). Insects, being small ectotherms, are especially vulnerable to environmental stressors such as temperature extremes, and a high surface area-to-volume ratio means that they are also at risk of desiccation (Gibbs et al. 1997; Gaston and Chown 1999; Hayward et al. 2004). The ability to tolerate both of these stresses is particularly relevant to species survival and distribution patterns in polar terrestrial environments (Convey 1996; Hayward et al. 2004; Convey et al. 2014).

### Environment and terrestrial habitats of Signy Island

At a latitude of 60°S, Signy Island is subject to the strong, circumpolar westerly airflow that surrounds the Antarctic continent. Combined with the winter expansion of Antarctic sea ice from the Weddell Sea that typically extends north of the South Orkney Islands, Signy experiences an annual climate that is more extreme than might be expected for its latitude, and comparable with that of Marguerite Bay more than eight degrees of latitude further south (Walton 1987; Hughes et al. 2013). However, within the South Orkney Islands, Signy does benefit from the warming effects of the Foehn winds that are drawn down from the mountains of the adjacent Coronation Island (King et al. 2017). As a result, Signy has positive summer monthly mean air temperatures between 0 and 3 °C and experiences microhabitat temperatures that can be easily in excess of 20 °C on the moss or soil surface, with spikes as high as 38.5 °C recorded (Walton 1982; Davey et al. 1992; Convey et al. 2018).

The ice-free areas of Signy Island are largely comprised of scree and moraine, with some areas of well-developed and diverse moss and lichen communities, as well as large moss banks and populations of the two Antarctic flowering plants, Antarctic pearlwort (*Colobanthus quitensis*) and Antarctic hair-grass (*Deschampsia antarctica*) (Smith 1972, 1990; Cannone et al. 2017). Vegetation is dominated by cryptogams, predominately turf and cushion forming mosses that are generally > 5 cm in depth (Smith 1972; Cannone et al. 2017), whilst soils on Signy, and in Antarctica in general, are typically thin with little humus content (Campbell and Claridge 1987). The depth and density of vegetation cover not only influences the formation of soils, but also microhabitat temperatures, acting as insulating blankets over the friable soil layer, keeping temperature more stable and preventing loss of water through evapotranspiration (Tenhunen et al. 1992). Typical annual precipitation on Signy is around 400 mm year^−1^ and occurs on 280 days per year on average, nowadays normally as rain in summer (Holdgate 1967; Walton 1982; Royles et al. 2013). Substrate moisture content on Signy is generally higher in summer, due to the combination of summer precipitation events in summer and melt of snow and ice (Gardiner et al. 1998; Bokhorst et al. 2007).

### Terrestrial invertebrate microhabitat selection and stress physiology

Antarctic terrestrial ecosystems present a challenging habitat for invertebrates, with low water availability from freeze and evaporative drought events, and low temperature seen as the two principle stressors (Cannon and Block 1988; Convey 1996; Block et al. 2009; Convey et al. 2014). Thus, microhabitat selection is a trade-off between maximising heat budgets for development and limiting the daily risk of desiccation or freezing (Hayward et al. 2003). Air temperatures on Signy can range by as much as 60 °C annually, from around − 40 °C to + 20 °C (Walton 1982), whilst the substrates in which *E. murphyi* is found also experience considerable temperature variation, with 21.8 °C diurnal fluctuation in the summer, ground temperatures below freezing in winter (Walton 1982) and an RH range of 37–100% (Worland and Block 1986). Bokhorst et al. (2008) reported that Signy soils have a greater number of summer freeze–thaw events than similar substrates on Anchorage Island, at c. 68°S off the Antarctic Peninsula, and Convey et al. (2018) report a longer delay in spring warming (a period when ground temperatures remain close to 0 °C during spring) on Signy than at other Antarctic sites, both in the maritime and continental Antarctic. Adverse temperature or moisture conditions can be alleviated to some extent through microhabitat selection, and there is clear evidence from other terrestrial invertebrates on Signy (Collembola and mites) that different thermal- and hygro-preferences reduce stress exposure (Hayward et al. 2001, 2003). However, in polar environments, there is typically limited refuge from environmental extremes, so the resident invertebrate fauna has had to evolve a range of stress response mechanisms. Amongst invertebrates, there are two basic physiological strategies to cope with desiccation stress: (1) Prevent water loss by being desiccation resistant; or (2) Tolerate the loss of water from the body by being desiccation tolerant (Danks 1999; Everatt et al. 2015). Previous work on the desiccation and heat tolerance of *E. murphyi* 4th instar larvae found that they are desiccation tolerant, and able to tolerate up to 46.7% water loss over 12 d, with little effect on survival (Everatt et al. 2014a). Furthermore, larvae can withstand temperatures of 39 °C for up to 1 h (Everatt et al. 2014c), whilst the larvae have received increasing research attention (Everatt et al. 2012, 2014a, b, c, 2015; Hughes et al. 2013) the eggs have been largely overlooked with no studies on any element of their physiology in over 25 years (Convey 1992). In this context, eggs are thought to only be laid during a short period in the brief Antarctic summer and, whilst sub-zero temperatures can still be experienced during this period, it is thought that low relative humidity or high microhabitat temperature extremes pose a greater risk to egg survival.

### Egg physiology and oviposition strategies

*Eretmoptera murphyi* reproduces parthenogenetically, laying single batches of 48–85 individual eggs within a large spherical hygroscopic gelatinous matrix, or egg sac, that has a water content of 96% when fully hydrated (Bartlett et al. in press; Cranston 1985; Convey 1992). Eggs take approximately 30 days to develop under summer field conditions on Signy Island (Bartlett et al. in press). This differs from *Belgica antarctica* (Jacobs 1900), the closely related endemic Antarctic midge, which produce 41 (median) eggs per batch, laid in a ‘ribbon’ arrangement, and which take 16 days to hatch at 4 °C (Harada et al. 2014). The investment in the single egg sac for *E. murphyi* is large, twice the dry mass of the post-oviposition female (Convey 1992) and may be the consequence of not adopting the multiple-oviposition strategy that typifies many terrestrial Orthocladiinae (Nolte 1993; Armitage et al. 1995). Producing a single egg sac appears a high-risk strategy, and so the hatching success rate of each sac might be expected to be high to compensate. However, under naturally fluctuating summer field conditions in the habitats to which it has been introduced on Signy Island, mean hatching success in field conditions is just 35% (Bartlett et al. in press). Similarly, low success rates were also recorded for *B. antarctica* at 4 °C under laboratory conditions (Harada et al. 2014). It is possible, though untested, that this may be a result of low fertilisation rates in *B. antarctica*, although this cannot explain low hatching success in *E. murphyi*, which is parthenogenic. Furthermore, Frouz (1997) notes that in chironomids the selection of a suitable oviposition site “affects the reproductive success of the whole next generation”, and thus could be strong selector on the survival of the eggs and, hence, the growth of the population.

Egg sacs of *E. murphyi* have previously been found in the surface vegetation layer in the vicinity of Signy Island Research Station (Convey and Block 1996). Whilst often highly saturated, this habitat is also prone to extremes in temperature and desiccation (Walton 1982). Considering the desiccation risk of such a habitat, Convey (1992) investigated the dehydration and rehydration tolerances of egg sacs, and found them able to tolerate short periods of extreme desiccation (26 h at 35% RH) by forming a ‘skin’ around the gelatinous matrix. Convey (1992) also noted that eggs tolerated temperatures above 10 °C and developed faster with increasing temperatures (over the range 2–12 °C). This is perhaps unsurprising given that chironomids typically have a development rate positively correlated to temperature, within optimal temperature boundaries following a hyperbolic law (Oliver 1971; Armitage et al. 1995; Frouz et al. 2002; Stratman et al. 2014). However, the temperature tolerance limits of *E. murphyi* eggs remain untested.

One strategy that can be adopted to reduce the impacts of temperature and desiccation stress in such habitats is to reduce the overall surface area-to-volume ratio of the egg sac, which can be achieved by producing eggs in clusters as well as by aggregating multiple egg sacs together. This latter strategy has been recorded in at least four species of Orthocladiinae as well as in many species of Chironomidae overall, including intertidal and alpine species (Armitage et al. 1995). However, communal oviposition is still apparently uncommon across the Chironomidae (Nolte 1993), particularly in terrestrial species that cannot rely on water currents to distribute hatched larvae and reduce intraspecific competition (Frouz 1997; Juliano et al. 2002). For brachypterous species, such as *E. murphyi*, adult dispersal ranges are certainly limited. In addition, parthenogenesis removes the need to seek out a mate. The challenge then becomes selecting oviposition sites in a landscape with a highly patchy distribution of favourable microhabitat conditions, whilst also limiting subsequent larval competition linked to high population densities.

### Aims of this study

Recent field observations made on Signy Island have confirmed that *E. murphyi* lays egg sacs both singly and in small aggregations (Bartlett et al. in press), but the oviposition sites have not been studied in detail. Thus, a primary objective of the current study was to determine if there is any evidence of microhabitat preference for oviposition sites in this species. Whilst the stress physiology of *E. murphyi* larval stages is well characterised, only one study to date has explored the stress physiology of eggs (Convey 1992). Here, we examine heat and desiccation tolerance limits of single egg sacs, as well as clusters of sacs, and place these in the context of microhabitat conditions encountered during summer on Signy Island. Finally, we correlate egg survival with environmental conditions experienced in different microhabitats and discuss the implications for the continued range expansion of this invading species under climate change.

## Materials and methods

### Sample collection and processing

**Fig. 1:**
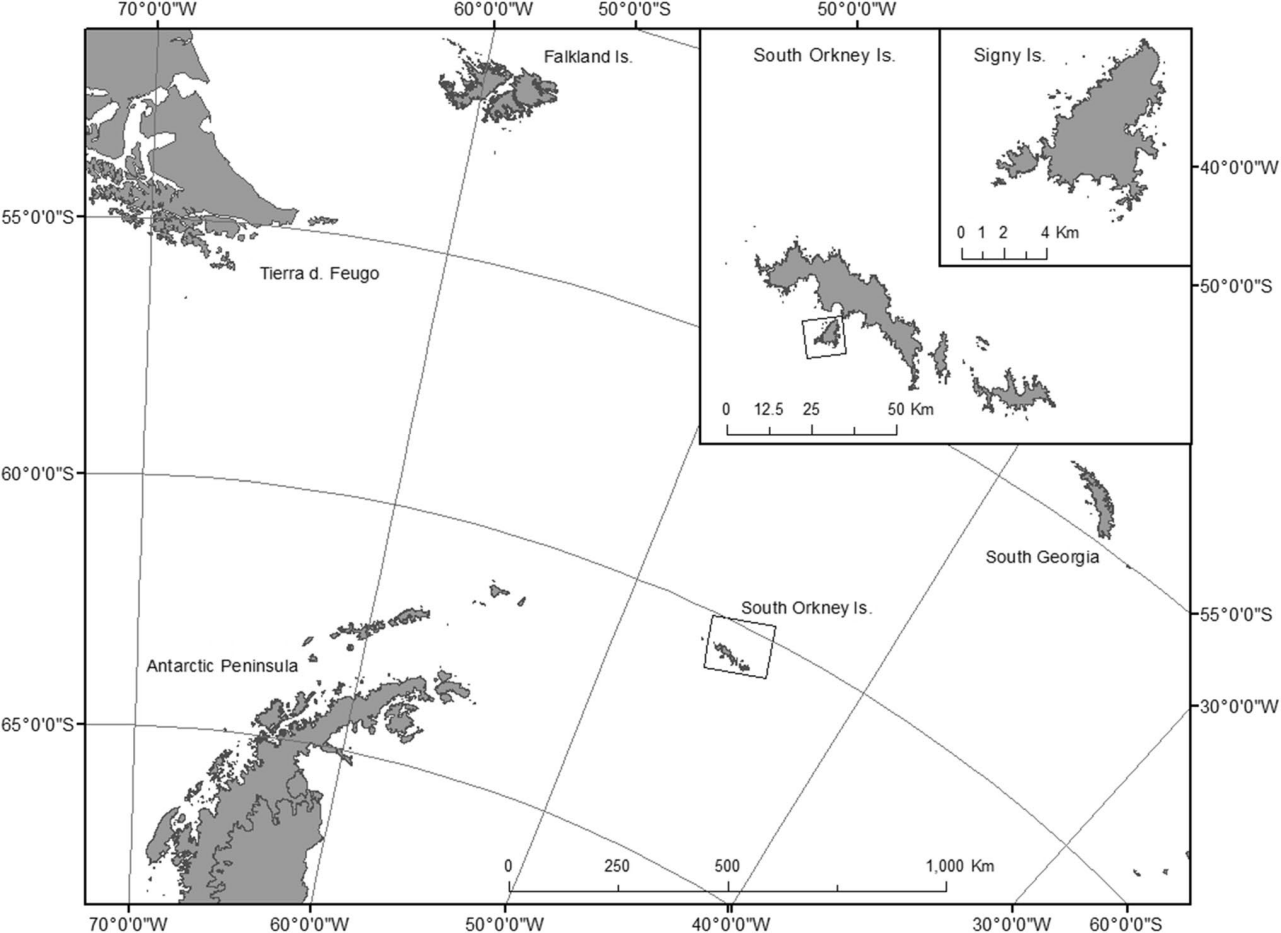
Location of Signy Island (South Orkney Islands) in the Southern Ocean. Created using ArcMap^®^ 10.4.1 software by Esri

All experiments were conducted in laboratories at the British Antarctic Survey’s Signy Island Research Station South Orkney Islands, maritime Antarctic, (60°43′0″S, 45°36′0″W) (Fig. 1) during January 2017, using recently laid egg sacs that had been collected from moss banks surrounding the research station. Egg sacs were obtained from the substrates with a Pasteur pipette and/or a paintbrush in order to minimise risk of damage to the sac and eggs contained therein. All eggs within the sacs were identified to be at the first (opal) developmental stage (Bartlett et al. in press; Harada et al. 2014) using a dissecting microscope (Leica EZ4). If any eggs showed signs of yellowing or embryonic development, the whole egg sac was discarded and not used in this study.

### Environmental data collection and substrate cores

Temperature and humidity loggers (Tinytag Plus II) were placed as a single logger ‘station’ at 5 cm below the surface (‘soil’), on the surface (‘ground’) and suspended 10 cm above the ground surface (‘air’). The loggers were programmed to collect data from the 16 December 2017 until the 21 February 2017, recording every 30 min. However, data logger failures meant that air temperature was recorded for 41 days, and ground temperature for 30 days, in total. Data presented (Table 1 and Online Resource 1) are from 12 Jan to 21 February 2017 when all loggers were working. To verify how close substrate saturation was to measured humidity, soil cores (*n* = 5) were taken weekly for 7 weeks from the 23 January 2017, to a maximum depth of 10 cm (where the underlying rock surface allowed) from a 1 m^2^ quadrat surrounding the temperature logger station. Cores were taken using a steel soil auger 2.5 × 10 cm, placed in individual sterile sample bags and immediately returned intact to the research station, where they were then separated into surface vegetation and peat/soil substratum layers. Total numbers of egg sacs were recorded for each layer and they were removed. Stones were also removed prior to the wet mass of each layer being recorded using a precision microbalance (Sartorius E-6202). Substrates were then dried at 60 °C to constant mass and weighed again to obtain dry mass. Water content was then calculated gravimetrically, as the percentage difference between fresh (wet) mass and dry mass.

**Table 1:** Mean, maximum and minimum air (at 10 cm); ground surface and soil (5 cm) temperature (°C); and mean, maximum and minimum relative humidity (%RH) at all locations

Substrate layer	*n*	Mean temp °C (± SEM)	Max. temp °C	Min. temp °C	Mean %RH (± SEM)	Max. %RH	Min. %RH	Mean % WC (± SEM)	Hours > 30 °C (% of total data)	Hours > 20 °C (% of total data)	Hours > 5 °C (% of total data)
Air (10 cm)	1376	5.2 ± 0.06	22.6	3.4	93 ± 0.4	100	< 10	–	0	1 (0.1%)	250 (36%)
Ground surface	1471	3.5 ± 0.15	31.2	− 4.3	81 ± 0.59	100	< 10	93.2% ± 0.5	2 (0.2%)	20.5 (3%)	176.5 (24%)
Soil (5 cm deep)	3102	2.5 ± 0.03	11.2	0.4	17 ± 0.1	29	< 10	71.1% ± 5.5	0	0	150 (9.7%)

Also shown: mean % water content (WC) relative to dry mass; and hours above experimentally relevant temperatures (30, 20 and 5 °C) for each ‘substrate’ layer

### Egg tolerance to heat exposure

To ensure that heat stress was not combined with desiccation stress, egg sacs were kept fully immersed in a medium similar to wet peat and moss turf. Immersion was not considered to be a stress as *E. murphyi* eggs are highly hygroscopic (Bartlett et al. in press; Convey 1992). ‘Field water’ was created by adding local substrate to deionised water at a 1:3 ratio. This mixture was then left for 1 week at 5 °C in the dark, to replicate the organic matter composition of the environment and provide the egg sacs with a relevant hydration medium that would provide a more even heat distribution than the use of soil substrate alone. The field water had a pH of 5.3 and a salinity of 221 µS, (comparable to measures taken from the moss bank the eggs were collected from—data not shown). Egg sacs containing only healthy ‘opal’ eggs were placed in groups of 10 into three 3-ml Eppendorf tubes with 1 ml of field water, giving a total sample size of 30 egg sacs per treatment. Tubes containing the egg sacs were placed into a water bath (Lauda C6) set to either 20 °C or 30 °C, and survival assessed after exposures of 2, 12, 24, 48, 96 or 192 h. Controls were held inside a 5 °C incubator. After exposure, egg sacs were assessed immediately for any signs of hatching, and then moved into separate petri dishes per treatment (no pooling), with local substrate that was saturated with field water. Petri dishes containing the exposed egg sacs were then kept at control conditions of 5 °C in constant darkness. Survival was re-assessed at 1, 3, 7, 14, 28 and 35 days post-exposure, using a dissecting microscope. Survival was quantified as the number of eggs that hatched successfully. After 35 days, the egg sacs were dissected and the total numbers of hatched and unhatched eggs, and the developmental stages reached by those that did not hatch, were recorded.

### Desiccation tolerance and water loss

Egg sacs were exposed to 100, 98.2 or 75% relative humidity (RH), at 5 °C, as either a group of 10 sacs (three replicates), or as a single sac (five replicates), to determine if clustering sacs together reduced rates of water loss. Prior to the experiment, the egg sacs (singly or as clusters) were weighed to the nearest 0.01 mg using a microbalance (Sartorius micro M3P). Egg sacs were then placed in petri dishes that were suspended above a solution of deionised water and NaCl using a nylon mesh fixed to the edges of the containers. The containers used were 2 l airtight plastic boxes, with one box per treatment. A relative humidity of 100% was obtained using pure deionised water (DH_2_0); 98.2% RH with 31.6 g NaCl/L DH_2_0, and 75% RH with saturated NaCl (Bayley and Holmstrup 1999). Temperature and relative humidity levels were monitored using data loggers (TinyTag Plus II) prior to each experiment’s initiation. Once humidity had stabilised at the desired RH, the egg sacs were placed into the container. All experiments were conducted within a 5 °C dark incubator. Egg sacs were exposed for 2, 12, 24, 48, 96 or 192 h and then removed, reweighed to measure water loss, and placed into control conditions, with subsequent survival assessed as described above.

### Statistical analyses

The relationships between time, survival and water loss in experimental studies, as well as microhabitat temperature and % RH data collected from environmental loggers across the summer season, were assessed using Spearman’s rank-order correlation, followed by further interrogation using Kruskal–Wallis tests. Spearman’s correlation was chosen over Pearson’s as the data were not normally distributed (D’Agostino and Pearson normality test) and environmental data, in particular, were deemed to be data with a monotonic relationship. Linear and Gaussian regression models were used to reflect the linear or non-linear relationships, respectively, at 95% confidence. In order to determine any difference in oviposition site choice between soil and moss layer over time, a Wilcoxon matched-pairs test was conducted after a normality test (as before). A Mann–Whitney test (unpaired) was used to compare the overall effects of egg sac clustering on water loss and % survival. The time at which the sample experienced 50% mortality is expressed as LT_50_. Following Suemoto et al. (2004), the LT_50_, water loss at 50% survival (WL_50_) and water loss rate (WLR) values were used as “measures of desiccation tolerance, dehydration tolerance, and dehydration resistance, respectively”. All calculations were conducted using the statistical software Prism 7.03 (GraphPad Software, La Jolla California USA). All survival data were corrected to the control data using Abbot’s Correction (Abbott 1925) prior to analysis.

## Results

### Environmental data

**Fig. 2:**
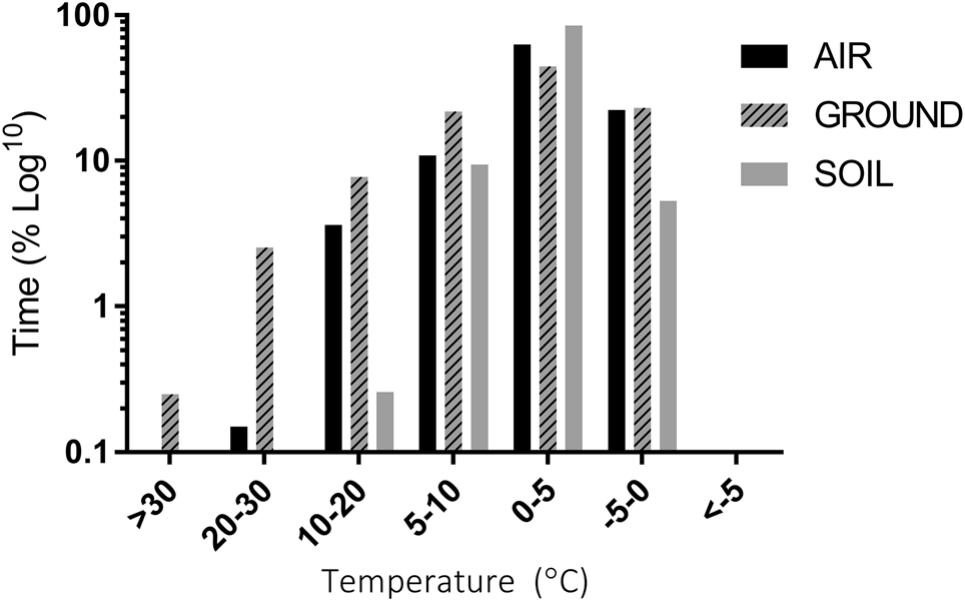
Percentage of time (log_10_ scale), that each habitat (air, ground and soil) spent within different temperature ranges. Dates surveyed for each habitat are as follows: ‘Air’ = 40 days, from 12 January–21 February 2017; ‘Ground’ = 30 days, from 26 December 2016–25 January 2017; ‘Soil’ = 91 days, from 18 December 2016–21 February 2017. No habitat experienced temperatures below − 5 °C

The temperature and % RH profiles of each substrate layer were significantly different (Online Resource 1) (temperature profiles, Kruskal–Wallis *H*_3_ =* 286, p *< 0.001; humidity profiles, Kruskal–Wallis *H*_3_ = 4217, *p *< 0.001). The highest temperature recorded was a ground surface temperature (GST) of 31.2 °C on the 12 January. Temperatures exceeding 30 °C accounted for 2 h of total recorded time (0.3%) (Table 1). GSTs exceeding 20 °C accounted for over 20 h of recorded temperatures (3%), with eight consecutive hours above 20 °C occurring on 12 January 2017. The lowest recorded GST was − 4.3 °C, on 9 January 2017. Figure 2 shows the overall time (h) spent within temperature brackets from 30 °C to − 5 °C, for all substrate layers.

**Fig. 3:**
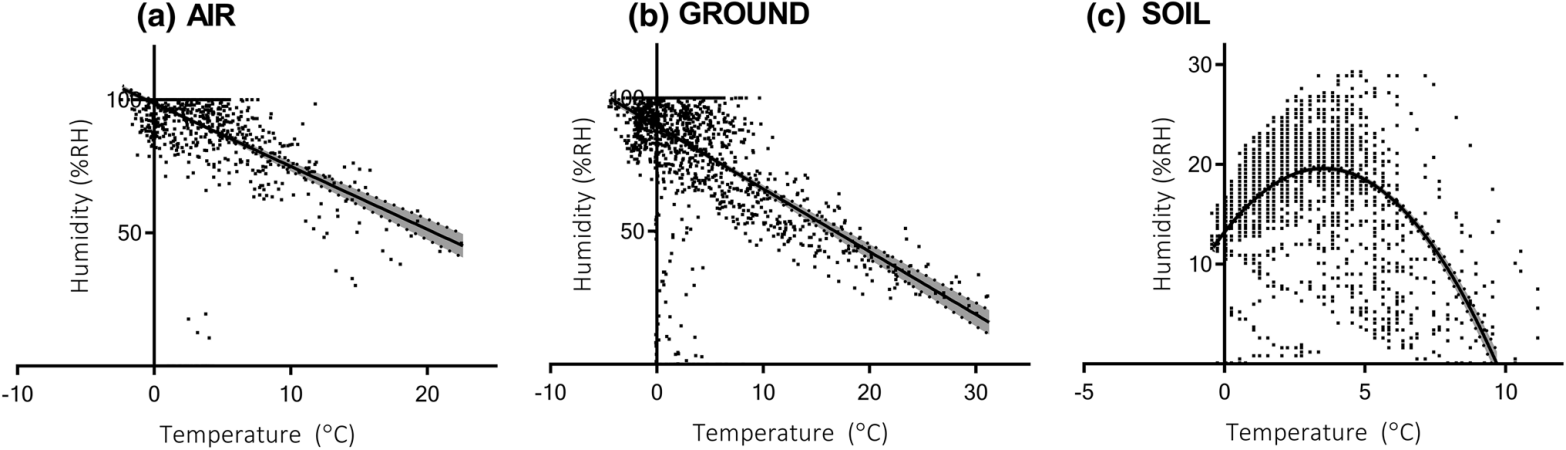
Temperature versus relative humidity for **a** air, **b** ground and **c** soil microhabitats (see Fig. 2 for dates). Linear regression plotted for air and ground; non-linear (quadratic) plot for soil (all shown with 95% CI)

Air and ground surface  % RH declined significantly with increasing temperature (Spearman’s rank correlation, *r*_*S*_ = − 0.54, *n * = 1376, *p* < 0.001 and *r*_*S*_ = − 0.46, *n * = 1471, *p* < 0.001 ,respectively; Fig. 3). Within the soil substrate, the relationship between % RH and temperature was non-linear (Fig. 3) increasing with temperature up to approximately 5 °C and then declining with any further increases in temperature (*r*_S_ = 0.33, *n * = 3102, *p* < 0.001).

Measurement of substrate water content over 7 weeks showed that the surface substrate, which is comprised of mostly moss turf, had a stable water content of between 90% and 97% saturation, with a mean of 93.2 ± 0.5% SE (*n *= 7) (Table 1). The soil layer had a more variable water content of between 46.8% and 87.1% with a mean of 71.1 ± 5.5% SE (*n * = 7).

### Oviposition sites

**Fig. 4:**
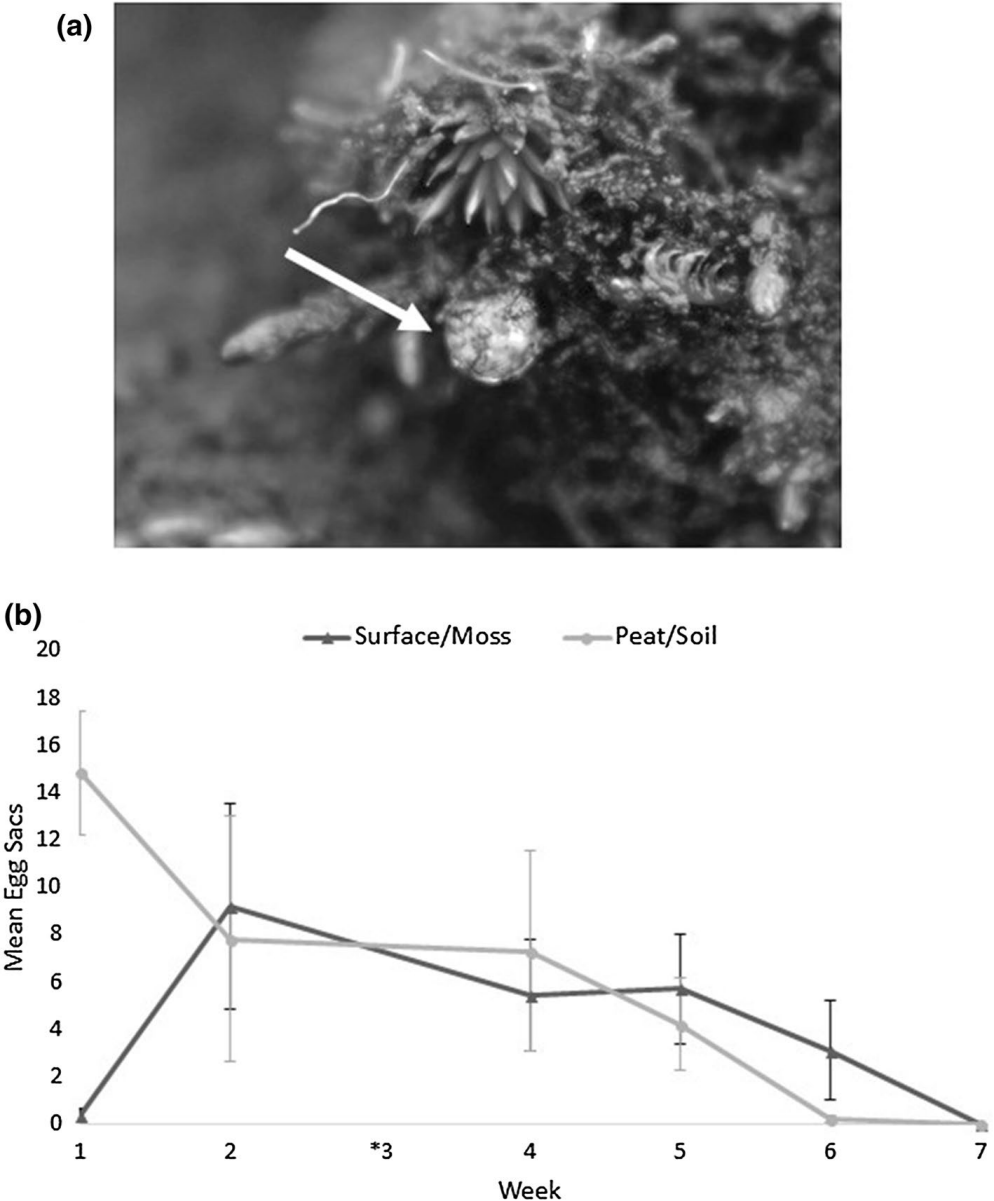
**a** Single *E. murphyi* egg sac laid on exposed surface vegetation. Image taken in situ, Signy Is. January 2017. **b** Mean ± SEM egg sacs found in each substrate layer over 7 weeks (*n *= 5 replicate cores), with week 3 (6th Feb) not shown as the cores were compromised prior to processing. No difference between the mean egg sacs oviposited in the two substrates over the period (Wilcoxon, *p *= 0.9)

Egg sacs were laid in both the ground surface vegetation and the soil substratum throughout the seven-week sampling period, 23 January 2017–6 March 2017 (Fig. 4). There was no strong preference for either substrate as an oviposition site (Wilcoxon matched-pairs test, *p *= 0.95), although there were initially more egg sacs in the soil at the beginning of the sampling period.

### Egg tolerance to heat exposure

**Fig. 5:**
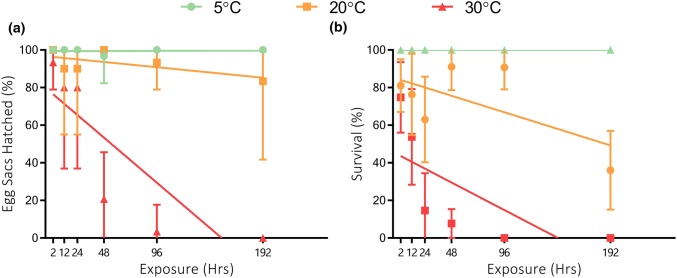
**a** Mean ± 95% CI %egg sacs (*n *= 30) that produced hatchlings after exposure to temperatures of either 5 °C, 20 °C or 30 °C, over periods of 2–192 h. **b** Mean ± 95% CI  %survival of individual eggs within each egg sac. Lethal time threshold for 50% of population (LT50) highlighted with a dotted line. Three replicates of 10 eggs sacs were used for each time-point at each treatment giving a mean value of *n* = 175 ± 28 SD individual eggs for 5 °C; *n* = 177 ± 20 SD for 20 °C; *n* = 187 ± 16SD for 30 °C. Friedman’s ANOVA, *F*(9.3) shows *p* = < 0.005 significance between the control (5 °C and the treatments of 20 °C and 30 °C)

A lower proportion of egg sacs produced hatchlings when exposed to the treatments of 20 °C and 30 °C compared to the control of 5 °C, with hatchling proportion decreasing over time (Fig. 5a). Overall, the difference between the three temperature exposures was significant (Kruskal–Wallis *H*_3_ = 9.9, *p *= 0.034) with the effect of 30 °C being the greatest (Linear regression, 30 °C = − 0.54 ± 0.15, *F*(_1,4_) = 9.69, *p *= 0.03; 20 °C = − 0.07 ± 0.03, *F*(_1,4_) = 2.85, *p * = 0.16; 5 °C = 0.0006 ± 0.008, *F*(_1,4_) = 0.03, *p *= 0.85).

Individual egg survival at 20 °C and 30 °C was significantly lower than for eggs maintained at 5 °C (Fig. 5b, Kruskal–Wallis *H*_3_ = 11.94, *p * ≤ 0.001). Survival decreased rapidly at 30 °C dropping to 60% within 12 h and reaching the LT_50_ between 12 h and 24 h. Survival was higher at 20 °C, and the LT_50_ was not reached over the 192-h exposure period.

### Egg sac desiccation rate and dehydration tolerance

**Fig. 6:**
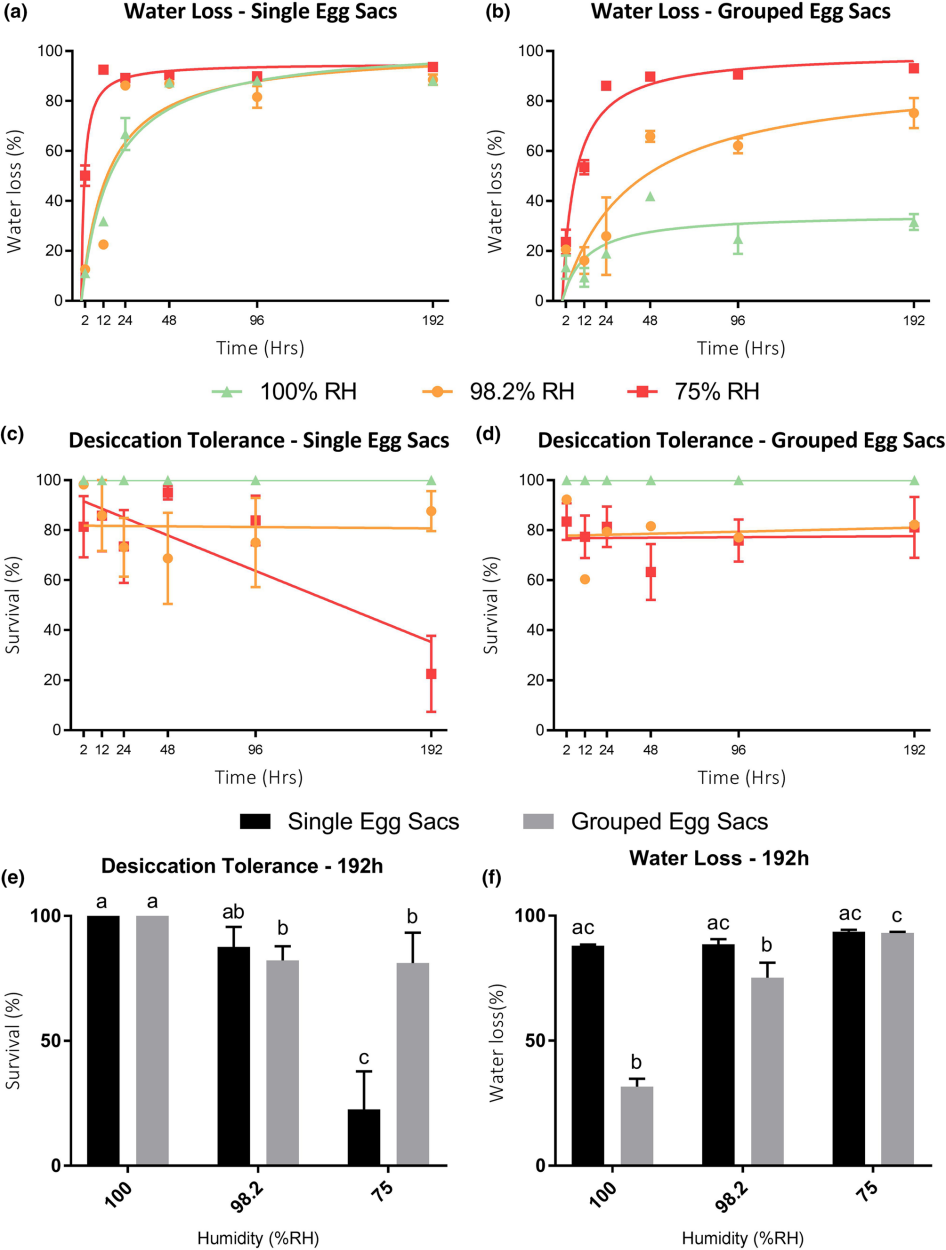
**a** Mean water loss (WL % relative to fresh mass) ± 95% CI of single egg sacs held under 100, 98.2 and 75% relative humidity conditions for 2–192 h. **b** The same data for *n* = 10 grouped egg sacs. **c, d** Mean  %survival ± 95% CI of eggs under same treatments with single and grouped egg sacs, respectively. **e, f** Mean water loss (%) and survival (%) ± 95% CI, at the maximum exposure time of 192 h. Graphs show the different response to humidity treatments between single and grouped egg sacs. Columns with the same letter are not significant (Kruskal–Wallis, alpha 95%). Treated and examined eggs sample size—Single egg sacs *n* = 5 (1 × 5 replicates), with a mean number of individual eggs at each exposure time -point of *n* = 255 ± 65SD for 100% RH; *n* = 241 ± 46SD for 98.2% RH; *n* = 237 ± 65SD for 75% RH. Grouped egg sacs, *n* = 30 (10 × 53 replicates) exposed at every variable *X* time-point, *n* = 12 of those randomly selected for dissection and examination. Of those 12: *n* = 864 ± 69 SD for 100% RH; *n* = 841 ± 74 SD for 98.2% RH; *n *= 807 ± 61 SD for 75% RH

All treatments resulted in a significant change in water content over time (Table 2) with an overall significant difference in water loss between single and clustered egg sacs (Mann–Whitney *U *= 1462, *p *< 0.001). Desiccation rates for single egg sacs were fastest at 75% RH, with 50% water loss within the first 2 h, and > 85% water loss after just 2 day (Fig. 6a). Water loss rates were slower (and roughly equivalent) at 98.2% and 100% RH, not reaching ~ 85% water loss until around day 4 (Fig. 6a). Desiccation rates for clustered egg sacs were again fastest at 75% RH, and without the initial rapid loss compared to single egg sacs, there was a much clearer distinction between rates of water loss at 98.2% vs. 100% RH (Fig. 6b). Clustered egg sacs did not reach 85% water loss until day 2 at 75% RH, after which water content stabilised (Fig. 6b). Water loss at 98.2% RH reached 60% around day 2, but never reached more than 70% across the entire 8-day experiment. Apart from one anomaly at 12 h (mean 27% ± 7.5 SE, *n *= 10), water loss in the 100% RH clustered samples never exceeded 30% (Fig. 6b).

**Table 2:** Spearman’s rank correlation matrix comparing time (h), %water loss (WL) and %survival

Correlation factors	Egg Sac clustering	100% RH	98.2% RH	75% RH
Time versus %WL	Single	0.87****	0.74****	0.52***
	Grouped	0.65**	0.77****	0.96****
% Survival versus time	Single	0	− 0.10	− 0.42*
	Grouped	0	− 0.04	− 0.09
% Survival versus %WL	Single	0	− 0.22	− 0.28
	Grouped	0	− 0.35	− 0.16

*R*_s_ values < 0.40 (moderate–strong correlations) are highlighted in bold

Significance of correlation denoted with asterisk: *p ≤ 0.05, **p ≤ 0.01, ***p ≤ 0.001, ****p ≤ 0.0001

The correlation of survival with time in single egg sacs was influenced by decreasing humidity, with the strongest relationship shown at 75% (Table 2). Single eggs sacs showed an overall decline in survival at all treatment humidities, but only reached LT_50_ between 96 and 192 h at 75% RH (Fig. 6c). Survival for clustered egg sacs did not demonstrate any overall declining trend across all humidity treatments (Table 2), but both treatments expressed lower survival overall (Fig. 6d) Despite an overall significant difference in water loss between clustered versus single egg sacs (Mann–Whitney *U* = 1462, *p* < 0.001) this was not reflected in overall  % survival difference (Mann–Whitney *U* = 5001, *p *= 0.07). However, at the maximum treatment time of 192 h, there was a difference in survival between 100% RH and 75% RH for both clustered and single egg sacs (Fig. 6e) (Kruskal–Wallis *H*_3_ = 10.2, *p *≤ 0.01 for single; *H*_3_ = 10.2, *p *= 0.01 for clustered), as a result of the increased water loss for clustered egg sacs at 75% RH (Fig. 6f) (Kruskal–Wallis *H*_3_ = 7.2, *p *= 0.02).

## Discussion

Terrestrial microhabitat conditions recorded on Signy Island during this study (Table 1) clearly indicate that the invertebrate fauna can experience temperatures in excess of 20 °C, as well potentially desiccating relative humidity conditions, during a typical summer. Whilst extreme temperature events are still rare on Signy, it is likely that they will increase in frequency under current global warming scenarios. The highest standard meteorological air temperature in Antarctica, 19.8 °C observed on 30 January 1982 (King et al. 2017), was recorded on Signy. This event came about as the result of an unusual band of high pressure from the South Atlantic Sector combined with the more commonly occurring Foehn winds that flow over Signy from the adjacent Coronation Island and have a warming effect. Foehn winds not only bring warm air, but also decrease the humidity (Speirs et al. 2010). Our microhabitat correlations between temperature and humidity (Fig. 3a, b) suggest that even slight increases in mean temperature could dramatically reduce air and ground surface humidity conditions, and moisture availability has been demonstrated as a key driver influencing terrestrial communities on Signy (Block and Harrison 1995; Kennedy 1995; Convey et al. 2003; Convey and Smith 2004). Interestingly, soil moisture content/humidity also appear to be highest at around 3–5 °C (approximately the current summer mean soil temperature on Signy), but declines rapidly as temperatures increase towards 10 °C (Fig. 3c). Mid-range climate forecasts for the Antarctic Peninsula and Scotia Arc region, including Signy and the South Orkney Islands, predict an average 1.5–2 °C warming by 2100 (Collins et al. 2013; Turner et al. 2014), and here we discuss the potential impact of these warming trends on egg survival of the invasive species *E. murphyi*.

### Eretmoptera murphyi egg temperature tolerance

Eggs exposed to 20 °C did not experience any significant declines in survival, even after 192 h of exposure (Fig. 5). This implies that the current warmest conditions on Signy (GST at or above 20 °C not exceeding 8 h) should not be detrimental to egg survival. Convey (1992) found that egg development rates in *E. murphyi* were positively correlated with temperature in experiments testing temperature regimes up to 12 °C but did not investigate specifically whether increasing temperature systematically influenced hatching success. Based on our laboratory data, *E. murphyi* eggs will also be able to tolerate the acute extreme events of > 30 °C GST recorded during the 2016/17 season, which lasted less than 1 h.

Climate forecasts for the region (Collins et al. 2013) suggest that mid-term temperature increases are unlikely to affect egg survival. As noted in other studies, the Antarctic terrestrial fauna is generally well adapted to the wide variation in temperature that often characterises their microhabitats and hence is not likely to be challenged—and may even benefit—from mean temperature increases of the scale predicted over the next century (Convey 1996, 2011). However, the forecast increase in the strength and frequency of Foehn winds throughout the whole Antarctic Peninsula sector (Cape et al. 2015; Turton 2017), and an increasing incidence of high pressure events from the South Atlantic sector (Turney et al. 2016), may result in more frequent and severe extreme surface warming events affecting locations such as Signy Island, with negative consequences for the terrestrial fauna, including an increased risk of desiccation. Temperature variation between the air and ground surface is dependent on infrared absorbance, with bright, clear days producing peaks in GST as sunlight heats the ground surface, which can be considered ‘extreme events’ influencing the contained biota. Overcast days, which are typical of the northern maritime Antarctic, especially the South Orkney and South Shetland Islands, may have warmer air temperatures but do not result in peaks in GST which, as found here, could have a lethal effect on egg development. Guglielmin et al. (2008), in a full year study covering a range of sites with multiple aspects on Signy, reported that sites with a northern aspect, such as the Backslope where *E. murphyi* is present, are more influenced by incoming radiation in the summer than the air temperature, thus more susceptible than other aspects to extreme surface warming. These features are evident in the data obtained here, with GST spiking above 30 °C on occasion and spending extended periods above 20 °C, even though ‘air’ temperature was at least 10 °C lower during these events. This temperature discrepancy was found even though the ‘air’ measurements presented here were taken within the GST boundary layer as defined in standard meteorological assessments (WMO 2008), and a more standard measurement of air temperature (at a height of 1.25–2 m) would show cooler temperatures and a larger discrepancy still (Walton 1982) but would be less applicable to the scale of *E. murphyi*. Convey et al. (2018) further highlight the importance of studies of GST and low-level air temperature when studying polar environments.

### Desiccation tolerance, egg sac aggregation and habitat choice

Relative humidity (RH) conditions recorded in terrestrial habitats on Signy spanned < 10% to 100% (Table 1) and, although conditions of 75–100% RH predominated at and above ground surface, the soil substratum peaked only at 29% RH (Online resource 1, Table 1). However, average available water content (WC) was measured at 71% and 93% of dry mass for the soil and surface substratum, respectively, suggesting that gravimetric assessment is a more accurate reflection of microhabitat saturation, at a scale relevant to invertebrates. We infer that air pockets surrounding the humidity sensor as a result of product design may artificially depress the relative humidity of the immediate environment, or be too large to measure experienced humidity at a scale relevant to *E. murphyi*. Previous studies on Signy have deployed various techniques to measure substrate moisture with limited success. Using volumetric measurements, Bokhorst et al. (2007) recorded soil moisture levels not exceeding 0.1%, whilst relative water content measurements of 100% were recorded by Royles et al. (2013) in comparable moss layers. Worland and Block (1986) reported RH readings of 37–100% in similar substrates, which are more consistent with results discussed here. Methodology aside, this study did find a high level of patchiness in soil saturation compared to the ground surface vegetation layer, with variability through time as well as across replicate substrate cores. Despite the ground surface vegetation layer acting as a sponge, and typically preventing the evapotranspiration of water from the soil layer below (Tenhunen et al. 1992), the gradient of the Backslope site combined with the permeable frost-shattered rock beneath (Matthews and Maling 1967) may drive the loss of water in the soil substrate. We suggest that this variance in soil saturation could underlie the patchy distribution of *E. murphyi* larvae as reported by Hughes and Worland (2010) and Bartlett et al. (in press) and even the varied reports in previous substrate moisture assessments (Worland and Block 1986; Bokhorst et al. 2007; Royles et al. 2013).

Previous research on soil invertebrates has clearly demonstrated that conditions as high as 98.2% RH, close to the wilting point of plants, can result in desiccation of species with permeable integuments (Bayley and Holmstrup 1999). This is certainly true of *E. murphyi* and *B. antarctica* larvae (Hayward et al. 2007; Everatt et al. 2014a). Importantly, however, the slower desiccation rates afforded by these RH conditions permit survival of > 75%, % water loss in *B. antarctica* and 46.5% loss in *E. murphyi* larvae (Benoit et al. 2007; Hayward et al. 2007; Everatt et al. 2014a). We find here that *E. murphyi* egg sacs also have poor desiccation resistance and lose water rapidly from the gelatinous matrix surrounding the eggs (Fig. 6a), but can be highly dehydration tolerant if rates of water loss are slowed, such as through sac aggregation (Fig. 6b).

Our data demonstrate that egg sacs oviposited in isolation experience much higher rates of desiccation, losing 50% of their water content in just 2 h at 75% RH, compared to around 20% water loss for clustered egg sacs over the same period (Fig. 6). This in turn affects survival, which fell below 50% within 96–192 h for single egg sacs, whilst no clustered egg samples reached 50% mortality even after 8 days at 75% RH (Fig. 6c, d). Water loss at 100% RH suggests that, in order to maintain maximum water content, egg sacs potentially need to be periodically submerged, or in contact with wet substrate. Whilst *E. murphyi* is not considered an aquatic species, its larvae do have the capacity to respire underwater and tolerate prolonged submergence (Everatt et al. 2014b). Thus, it could be argued that they are semi-aquatic/terrestrial given the saturated moss banks in which they often reside (Convey 1992). This dependency on access to water for egg sacs is reduced, however, if they are clustered together—presumably because a reduced surface area slows water loss rates from the sac matrix. Clustering of egg sacs from multiple females during oviposition could, therefore, be a behavioural adaptation which has facilitated the transition of *E. murphyi* to a more terrestrial life style.

Whilst the compounding effects and potential role of cross-tolerance between heat and desiccation stress responses has not been explored in this study, previous work on polar chironomids has produced varied results. Everatt et al. (2014a) reported that prior exposure to desiccation at 98.2% RH had no effect on the heat tolerance of *E. murphyi* larvae at 30–40 °C. Pre-exposure to 0, 75 and 98.2% RH, in contrast, improved the heat tolerance of *B. antarctica* larvae at 30 °C (Benoit et al. 2009). There is also evidence that desiccation improves survivorship at low temperatures across not only *E. murphyi* (Everatt et al. 2014a) and *B. antarctica* (Benoit et al. 2009), but also other polar invertebrates such as the dipteran *Heleomyza borealis* (Everatt et al. 2014a) and the springtails *Folsomia candida* (Holmstrup et al. 2002) and *Cryptopygus antarcticus* (Elnitsky et al. 2008; Everatt et al. 2013). This positive relationship is the consequence of similar injuries resulting from low temperature and low water availability, and the same physiological processes being activated in response (Bayley et al. 2001). Such features being expressed in eggs would allow them to continue hatching after a sudden summer freeze event where both water availability and low temperatures are experienced. But the combined effects of drought and increased temperatures may be detrimental considering the findings of dual stress response in *E. murphyi* larvae (Everatt et al. 2014a).

Mean %RH for different *E. murphyi* oviposition sites, i.e. surface vegetation and within the soil (Table 2), suggest that the latter environment would expose single egg sacs to conditions that impact on survival. The relationship between soil humidity and temperature (Fig. 3c) also illustrates that the soil substrate is susceptible to desiccation under both cooling (< 3 °C) and warming (> 5 °C), whereas the surface vegetation is consistently wetter/has higher  %RH at lower temperatures (Fig. 3b). Bokhorst et al. (2007), also found a non-linear relationship between soil moisture and temperature, and that small increases in temperature led to rapid decreases in soil moisture. This makes the soil habitat a highly variable environment, with less favourable RH conditions than the surface vegetation that is dominated by water-retentive moss turf, and where GST conditions afford higher thermal budgets for development without risking survival. The disadvantage of clustered oviposition could be increased competition for resources once eggs hatch. In this regard, it is interesting to note that chironomid midges do not generally produce egg batches or sacs in a clustered fashion, particularly terrestrial species (Nolte 1993; Armitage et al. 1995). However, there is currently no suggestion of a food shortage for detritivorous *E. murphyi* larvae within their habitats on Signy Island, and aggregation likely generates more favourable conditions, as has also been proposed for microarthropods in the maritime Antarctic (Cannon and Block 1988; Block and Convey 1995; Hayward et al. 2004; Schulte et al. 2008).

## Conclusion

Oviposition site selection, whether in soil or on the surface vegetation layer, has important implications for environmental conditions experienced by eggs—with pros and cons for each microhabitat: in the soil, whilst measurable %RH is typically low at the spatial scale of our logging equipment, actual water content can be high within the substrate itself, albeit with a patchy distribution depending on topography and underlying geology. Soil temperatures on Signy are typically cool and stable, but the thin soils mean small changes to this can greatly affect water content. Within the north-facing site where *E. murphyi* were sampled, the ground surface layer is much more sensitive to irradiation and experiences large spikes in temperature, but also has higher mean %RH than within the soil. Given these microhabitat differences, and the vulnerability of egg sacs laid singly to desiccation, we conclude that oviposition within the soil is a higher risk strategy unless in direct contact with a continuously saturated substrate. This may explain why soil oviposition was higher early in the season where it would coincide with spring thaw. It is unusual for terrestrial chironomids to lay egg sacs in aggregations, however, *E. murphyi* does employ this strategy, and we have shown that egg sac clusters are able to survive in both soil and surface vegetation habitats, and that aggregated oviposition could be an advantageous behavioural strategy. Current temperature patterns and extremes on Signy Island are unlikely to affect this species survival regardless of oviposition site but predicted changes particularly in the frequency and duration of extreme events with continued climate warming and changing precipitation patterns are likely to challenge egg batch survival.

## Electronic supplementary material

Below is the link to the electronic supplementary material.

Supplementary material 1 (PDF 150 kb)
