# Supplementary material for: Not so free range? Oviposition microhabitat and egg clustering affects Eretmoptera murphyi (Diptera: Chironomidae) reproductive success
Source: Polar Biol. 2018 Oct 16;42(2):271–84. doi: 10.1007/s00300-018-2420-4 (PMC6383618; doi:10.1007/s00300-018-2420-4)
Supplement: Supplementary file 1 — Supplementary material 1 (PDF 150 kb) [file 300_2018_2420_MOESM1_ESM.pdf]

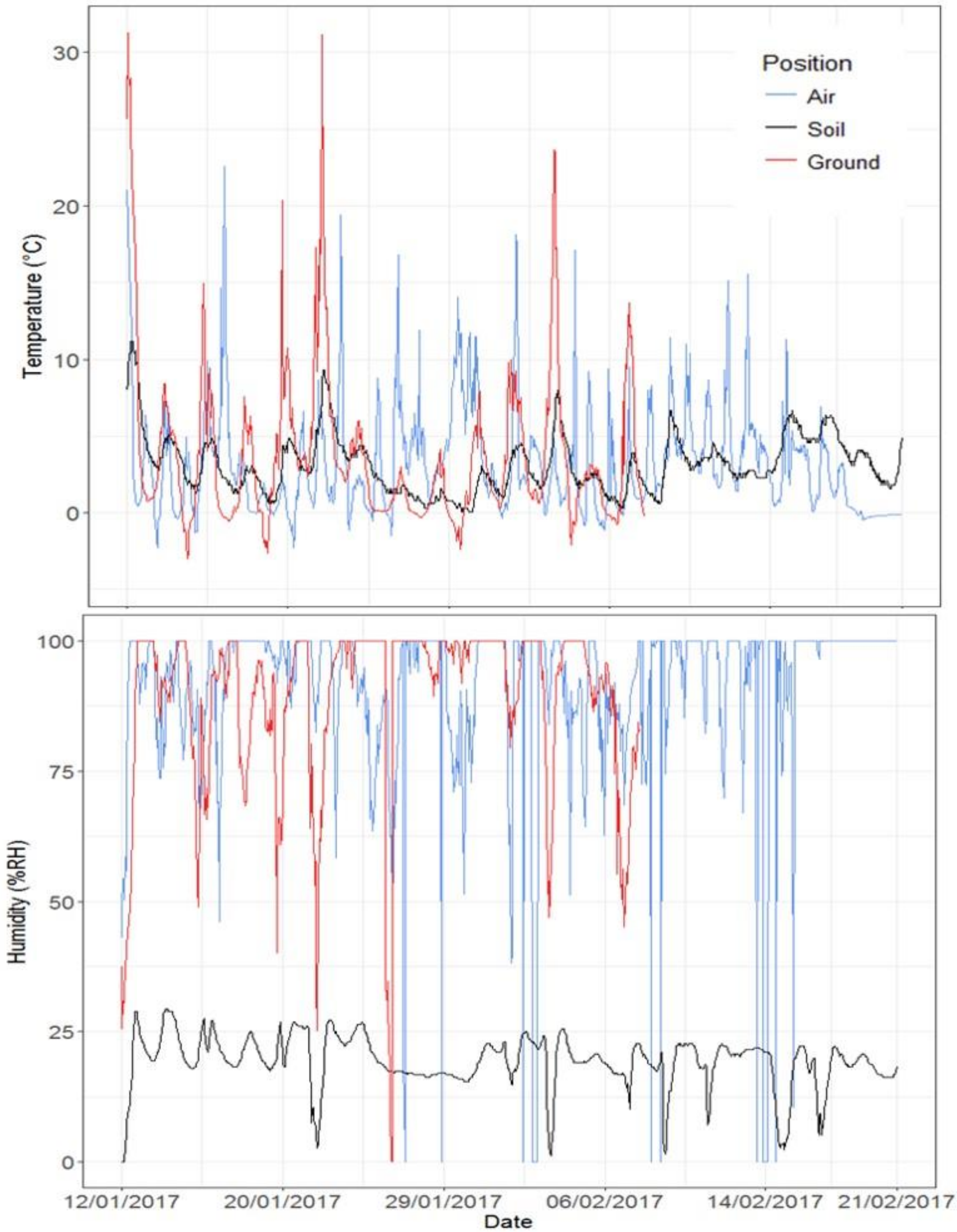

Online resource 1 Air (10cm above ground), Soil (5cm below surface) and Ground/surface, temperatures and relative humidity recorded on Signy Island in *E.murphyi* habitat 12th Jan to 21st Feb (40 days)
